# Shut up, or Set Free: Poetic Inquiry into Disabled Students’ Experiences of Differential Attainment

**DOI:** 10.5334/pme.1392

**Published:** 2024-11-21

**Authors:** Megan E. L. Brown, Gabrielle Finn

**Affiliations:** 1 School of Medicine, Newcastle University, UK; 2 University of Manchester, UK

## Abstract

**Introduction:**

Differential attainment (DA) – systematic differences in training and assessment outcomes when grouping individuals by demographic characteristics – is a pervasive problem in health professions education. Despite evidence of its prevalence, there have been few qualitative studies relating to disabled learners’ experiences of differential attainment. This represents a significant gap, as understanding disabled learners’ experiences is key to developing effective interventions that mitigate the impact of differential attainment.

**Methods:**

We used critical poetic inquiry to explore the lived experiences – including emotional, cultural, and contextual dimensions – of differential attainment for disabled health professions students. We constructed poems following a secondary analysis of a large interview dataset (n = 123 participants) from one institution. We focused on students who disclosed disability (n = 18), narrowing to health professions education (n = 10).

**Results:**

Poems reflect individuals’ experiences of DA. Four themes were constructed, within which we use poems to illustrate key connections: The perseverance stereotype, Managing the hidden curriculum, Privilege and access, and Surviving, not thriving. These themes illustrate the complex interplay of systemic barriers, ableist stereotypes, and privilege in the educational journey of disabled students.

**Discussion:**

The poems reveal the often-unseen struggles of disabled learners, challenging ableist perceptions and highlighting the necessity of inclusive practices. Our findings underscore the need for a shift in educational approaches, advocating for universal design and comprehensive support systems that consider the unique experiences of disabled learners. This study lays the groundwork for future research to develop interventions that address DA in a more inclusive and equitable manner, ensuring educational environments support all learners effectively.

## Introduction

“They shut me up in prose”. These are the words of Emily Dickinson, in a poem which has been titled as such. One interpretation of this rebellious statement relates to the censorship Dickinson experienced throughout her career from publishers dissatisfied with her creative use of spaces and dashes. By the end of the poem, Dickinson likens herself to a bird that has laughed at, and destroyed, its cage – she has found freedom in creative expression, in her unique approach to poetry, rather than traditional approaches to prose.

We have chosen to begin this manuscript by drawing reference to this wonderful poem because it captures a feeling we have shared as researchers interested in the experiences of disabled learners in health professions education. Just as Dickinson expressed confinement within the rigid structures of prose, we, too, have encountered the limitations of traditional academic reporting when attempting to convey the complex, nuanced experiences of disabled learners. The strict formats of prose often fail to capture the emotive depth and the textured lived experiences of disabled people. In adopting poetic inquiry, we can find new ways to vividly and viscerally portray the realities faced by these individuals.

This methodological shift can be of particular use when we consider differential attainment (hereafter, DA), a “wicked problem” in health professions education [1]. DA can be defined as “the gap between attainment levels of different groups” [2], or as systematic differences in training and assessment outcomes when grouping individuals by demographic characteristics [3]. Existing research has focused on establishing the presence and prevalence of DA – we know, for example, that for health professions students DA is more pronounced for students with minoritised identities and backgrounds – non-white medical students are 2.5 times more likely to fail high-stakes examinations compared to white students [4]. Disabled learners have rarely been a focus of conversations relating to DA, though we know they do experience DA e.g., in postgraduate surgical examinations [5], and at medical school [6].

However, much research on DA and disability has been quantitative. Ellis et al. [5] quantitatively reports disabled candidates scoring less well in written examinations within postgraduate surgical training, whilst Revell and Nolan [6] highlight differential grading at medical school. Similar patterns have been quantitatively reported in the United States, where medical students with learning disabilities are reported as less likely to graduate on time, less likely to match on first attempt at residency, and less likely to score highly on national examinations [7]. Whilst such quantitative work has been critical in establishing that disabled learners experience DA, there is a relative lack of qualitative exploration regarding disabled learners’ lived experiences of DA.

Much of the qualitative work we do have focuses on disabled learners’ lived experiences broadly, as opposed to disabled learners’ lived experiences of DA. Whilst focused work on DA is required, and constitutes the goal of this research, broader work on disabled learners’ experiences is relevant, as understanding the context of learners’ experiences is likely to provide important insights into barriers faced in educational settings. Within the UK, Shaw and Anderson’s body of research on the experiences of dyslexic learners highlights the emotional distress, stigmatisation, and lack of support students routinely encounter [8,9,10]. Within Canada, Jarus et al. [11] note regular challenges to disabled learners’ participation, including lack of transparency around competency evaluations, and exclusionary social and physical contexts. In the US, Jain’s [12] work on students’ experiences of disclosing disabilities highlights how some students engage in “political disclosure”, sharing their disability strategically to counter stigma and promote change. Collectively, these studies offer important, holistic insights on disabled learners’ experiences of medical education structures and environments. Fewer studies consider disability specifically in relation to DA. Although often disability is mentioned as an influential intersectional factor in relation to other minoritised identities (e.g., experiences of Widening Participation students [13]) it is yet to receive dedicated attention.

This is a critical gap in the literature – whilst the barriers disabled learners face, such as stigmatisation, are increasingly well-documented, how these experiences relate to DA has not, to our best knowledge, yet been considered qualitatively, through exploring lived experience. Whilst qualitative work cannot tell us statistically whether a learner has numerically experienced DA, we believe exploring students’ own thoughts and meaning making in relation to their experiences of academic performance, and barriers they may have faced, is still useful. Many students are now well-aware of DA [14,15], and by exploring disabled learners’ sensemaking we can examine how perceptions of fairness, support, and academic achievement intersect with their identities as future healthcare professionals. In addition, there is a strong likelihood that disabled learners have, or will, experience DA, extrapolating from existing quantitative investigations [5,6,7].

Failing to address DA means significant negative impacts both for the individuals DA affects, and for systems facing a workforce crisis, such as the UK National Health Service [16]. To adequately address a “wicked problem”, you need to first understand it, in all its complexity [17]. Exploring the experiences of disabled learners regarding DA is central to this, as is poetry as a creative method of analysis that can help us appreciate some of the complexities of these experiences. Through poetry, we can begin to unravel the web of factors contributing to DA, including the less tangible aspects of culture, identity, and personal experience that, in our experience as educators, play a crucial role. The aim of this research is to use poetic inquiry to explore deeply the subjective, emotional, and to-date relatively overlooked experiences of disabled learners facing differential attainment in health professions education.

## Methods

### Paradigm, and methodology

Poetic inquiry is an arts-based research method, which employs poetry for the interpretation and presentation of data. Approaches to analysis with the umbrella of poetic inquiry are diverse – what is key to ensuring quality and rigour is transparency of the rationale for use of the methodology, and of the approach taken [18]. We have chosen to adopt a critical approach to poetic inquiry [19], which is constructionist in paradigm [20] and focuses on exploring and naming underlying power dynamics, social structures, and cultural contexts that influence and shape the experiences of students in relation to differential attainment. This is appropriate, given our interest in embodying disabled students’ experiences of differential attainment.

### Dataset

We undertook a secondary analysis of a subset of an existing, large dataset on student and staff experiences of differential attainment at the University of Manchester. The dataset was collected through one-to-one semi-structured, one-hour interviews with staff and students, across university faculties. The original research guiding collection of this data was a realist evaluation of differential attainment within the university, considering factors such as race, gender, socioeconomic status, and departmental differences [21]. The interviews were designed to capture a broad range of experiences and perceptions, providing rich, qualitative data on how differential attainment manifests at the individual and group levels. Interview questions asked participants about their experiences of attainment, achievement, and failure, and how these intersected with their identities (See Supplementary Material for Interview Questions guiding dataset collection). There were 178 participants in total, 123 students, and 55 staff: with data collection taking place between 2022, and 2023. The original research has yet to be published.

Ethical approval was collected to facilitate secondary data analysis (Ref: 2022-15124-24932). Given we are interested in disabled students’ lived experiences, we chose to focus only on student data (n = 123) within this large dataset, to facilitate an in-depth analysis. We screened all student data for disclosure of disability status, selecting those where disability had been disclosed (n = 18). Given our context within health professions education, we also chose to narrow to students studying for a health professions degree (n = 10 in total).

### Constructing poems from this dataset as our analysis

We followed Glesne’s [22; described in 18] approach to poetic inquiry, adhering to the key principles, of: using only the participant’s own words in the creation of participant-voiced poems from their interview transcripts; paying close attention to participants’ use of language/grammar/syntax to emphasise authenticity; and embracing the flexibility to draw on language used across a participants’ transcript, in any order, to communicate layers of meaning within a transcript. Whilst Glesne’s [22] approach was developed for primary data collection and analysis, we chose to apply their principles to secondary data analysis, recognising the inherent flexibility and adaptability of poetic inquiry, as highlighted by numerous scholars (e.g., [23,24]). We have clearly outlined our process, below, in attention to methodological alignment with Glesne’s approach, and considered the limitations of applying this to secondary analysis in our discussion.

The aim of our poetic analysis was to explore the experiences of disabled health professions students in relation to DA. We were specifically attuned to the complexities of students’ journeys including the challenges they may have faced, and their successes, which may not have been the focus of the original realist evaluation. By employing poetic inquiry, our goal was to transform students’ detailed accounts into powerful accounts of how DA is lived by disabled students.

We began by familiarising ourselves with the dataset, conducting a detailed review of each transcript to understand participants’ use of language. We manually coded the transcripts, identifying significant phrases and sentiments, and made detailed memos. Regular meetings ensured we focused on both content and delivery, developing analytical codes that explored hidden meanings and power dynamics. Categories were created and edited to group codes, with MB leading on grouping categories into draft themes, and on returning to the data to incorporate unrepresented experiences. Once themes were agreed, we created poems for each transcript using Glesne’s approach. Poems were created by weaving together evocative language and metaphors, and were reviewed and refined for clarity, rhythm, and emotive impact as a team. Final poems were selected for their vivid representation of our themes, and we tried to ensure inclusion of poems from different participants, with varied experiences. MB wrote accompanying analytical commentaries to contextualise the poems, forming the text of the results report.

### Reflexivity

Given the deeply interpretative process of constructing participant-voiced poems, it is important we, as authors, reflect on our own experiences of differential attainment. Throughout, we engaged in continual shared consideration of how our own experiences and perspectives shaped our approach. In dialogue with one another, with our poems, and with our participants’ stories, we critically appraised and challenged our interpretations. Below, are statements from each researcher regarding how we engaged with our own contexts – our positionality – in this broader process of critical self-reflection.

Megan: I am multiply disabled, having become disabled whilst training at a postgraduate level as a doctor. I have very negative experiences of support within postgraduate training, which ultimately led to me making the decision to leave medicine. I bring this lived experience to this research, both as an interpretative lens, but also as a motivator for exploring the experiences of disabled learners. I have experienced discrimination, lack of access, and accommodations across my professional career, and have drawn on this experience in creating the poems in this study.

Gabrielle: I am a researcher and educational leader with an interest in equality, diversity, and inclusion. I have researched disability within differential attainment and clinical career progression. I have no disabilities but have first-hand experience of the challenges around disability and chronic illness as a parent. In the context of this study, I feel I bring both an understanding of the real-world impact of disability and an understanding of institutional support processes (and gaps) that have helped to contextualise findings and recommendations.

## Results

### Demographics

We created poems from the transcripts of 10 disabled health professions students. We have chosen to pool demographics, rather than assign these strictly to the poems created, or provide a line-by-line table, to preserve confidentiality. Where we feel poems cannot be interpreted by a reader without demographic context, we provide this in the results section, below.

All students disclosed a disability. We are not reporting specific disabilities here, due to risk of breaking anonymity. Disabilities spanned mental illness, neurodivergence, physical disabilities, and multiple disabilities. Average interview duration was 57 minutes. Both men and women were represented, but we had no participants identifying as other genders. Participants included both international students and those with home fee status.^1^ Ethnically, the participants represented a mix of backgrounds, including White British, Asian/Asian British, and Black/African/Caribbean/Black British identities. The health professions courses they were enrolled in spanned a range of health disciplines (Medicine; Nursing; Dentistry/dental hygiene; Medical biochemistry; Optometry), indicative of the diversity within health professions education.

### Overview

We created detailed codes through the in-depth exploration of 10 transcripts. These were organised into 10 high level categories, which are captured and connected by our four themes. We have assigned participants pseudonyms, to acknowledge that each participant is a person, not a number. Pseudonyms were selected based on participant gender and ethnicity using the website NameBerry [25] by the research team.^2^

Here, we present one to two poems per theme, which we feel capture the meaning of the themes, both for the student(s) whose poem(s) are presented, but also across many of the transcripts we reviewed. Our four themes are: 1. The Perseverance Stereotype; 2. Managing the hidden curriculum; 3. Privilege and access; and 4. Surviving, not thriving.

### Theme 1: The perseverance stereotype

Our first theme has been created in acknowledgment of the intense pressure placed on disabled students to conform to abled, neurotypical standards of success. These standards often operate under the false assumption that all it takes is more effort – or perseverance – to overcome any obstacle, including differential attainment. This leads to the manifestation of the belief that disabled students simply need to ‘try harder’ if they want to achieve the same outcomes as non-disabled students.

We feel this stereotype is typified by a poem constructed from Taha’s interview. Taha is neurodivergent and describes the ableist assumptions, or stereotypes, that disabled people just need to try harder to achieve when they experience differential attainment.

“If you try hard, you will do well”

I became strange
when all my symptoms
lined up.

I became forgotten
in huge competition,
lingering
in my head.

Not knowing how
to do well.
Tackling!
Forcing!
Struggling!

The impossible cost
of trying.

The use of words like “Tackling,” “Forcing,” and “Struggling” emphasises the physical and emotional toll of trying to conform to societal standards of success. These words convey a sense of exertion and resistance, highlighting the often-overlooked struggles that neurodivergent individuals face in their efforts to succeed in a world not designed for them.

Taha’s experience resonates with those of other disabled students in our dataset. Amelia, who is also neurodivergent, described feeling isolated from an educational system that fails to accommodate different ways of thinking and learning. Below, we share an excerpt from the beginning of the poem we created from her transcript, “Over a wall”, where we have tried to communicate the alienation Amelia expressed:

Over a wall

I am over a wall
they built.

I am behind
and below

and cannot see
what they see

but what they see is
“unsatisfactory”.

The wall in this poem symbolises systemic barriers that separate Amelia from success standards imposed by said system. Her struggle to “see what they see” reflects a disconnect between Amelia’s reality and the expectations placed on her, leading to her efforts being labelled “unsatisfactory.” The poem critiques the stereotype that perseverance alone can overcome obstacles – this belief ignores systemic failures that unfairly judge disabled students’ capabilities.

Similarly, the phrase “The impossible cost/of trying” in the poem created from Taha’s interview is a critique of societal expectations placed on disabled individuals. It underscores the idea that the demand for continuous effort and perseverance can be unreasonable and damaging, especially when it does not acknowledge the inherent challenges faced by disabled people.

Overall, the stereotyping that Taha and Amelia describe minimises the complex realities and obstacles faced by disabled students. Disability cannot be overcome through willpower – this stereotype is deficit orientated (i.e., it positions disability as something inherently negative that needs to be “fixed”) and ignores the pervasive and convoluted barriers that exist in educational, professional, and personal environments.

### Theme 2: Managing the hidden curriculum

This theme explores the implied expectations within higher education —what is commonly referred to as the “hidden curriculum.” For disabled students, particularly those who are neurodivergent or multiply disabled, navigating the hidden curriculum can lead to significant challenges. Students must constantly adapt to inconsistent standards, all while grappling with the systemic barriers that are often overlooked by those who dictate these standards. The hidden curriculum not only communicates academic expectations, but also tells learners who struggle to meet not only formal, but these implied standards of practice, that they are less capable.

Here, we present a poem constructed from Blake’s interview. Blake is multiply disabled and neurodivergent. This poem speaks to the contradictions between what is said, and what is done within universities – Blake’s experiences of the hidden curriculum of his environment.

Not this, but that.

Not dropped out, but thrown out.
Not silence, but noise.
Not passion, but overwhelm.
Not brief, but detailed.
Not guided, but indefinite.
Not enough, never enough.

Not accessible,
Not supported,
Not more than one thing.
Not me, but here.

We constructed this poem to communicate the contradictions in Blake’s experience, the uncertainty he expressed in terms of managing these and knowing how to move forward. For instance, the line “Not guided, but indefinite” was constructed in response to Blake sharing his experience of a lack of support in navigating academic content. Across the health professions, there is an incredible breadth of knowledge students could engage with or could learn. Blake described struggling to know where to find the “right” information, struggling with hyper-focusing on topics he was interested in at the expense of learning other required learning outcomes, and feeling overwhelmed by the sheer volume of content he was instructed to cover.

This represents the hidden curriculum in action – it is assumed that students will possess neurotypical study skills or be able to easily access the support to navigate a vast “indefinite” knowledge base. For neurodivergent students, this assumption can lead to challenges, as Blake expresses. The hidden curriculum – which, in the context of health professions education, is grounded in ableist norms – imposes an additional burden on disabled students; they must not only meet the formal academic requirements but also decipher and adapt to unspoken expectations, often without support.

### Theme 3: Privilege and access

Within this theme, we present a poem constructed from Jada’s interview. Jada is disabled and from an ethnically minoritised background. This is a short poem, purposefully, to communicate severe gaps in access to necessary support services, both for physical and mental health needs, for disabled students. The poem considers how existing services are navigated and accessed, and the significant role of knowledge and privilege within this.

Accessing support is …
Hearing a passed-down whisper,
or hearing nothing at all.

Given the intentional brevity of Jada’s poem, we present another poem on this theme, which we feel offers a different perspective. Zain is a student who is also a migrant, who spoke at length about his experiences with disability relating to mental health.

Starting, failing

I started,
                    Not keeping up,
I failed.
                    Not speaking up.
I started,
                    Living backwards,
I failed.
                    An old half.
I started,
                    Looking for help,
I failed.
                    Not able to talk.

“Just say the word!”
Just say the word.
But with everything,
I couldn’t.

The poem constructed from Zain’s interview, “Starting, failing”, is characterised by a repeated structure of “starting” and “failing,” symbolising a continual cycle of effort and setback. This repetition reflects the struggle faced by disabled learners, where attempts to seek help are often met with internal and external obstacles. The recurring line at the end of the poem “Just say the word!” juxtaposed with “But with everything, I couldn’t” captures a key paradox in Zain’s experience. While help often begins with communication, the act of expressing one’s needs can be challenging for disabled people. Compounding this for Zain may be language and cultural differences which he expressed in his interview make the act of “starting” and the experience of “failing” more pronounced and complex.

### Theme 4: Surviving, not thriving

In our final theme, “Surviving, Not Thriving,” we explore how, despite accessing formal support services, disabled students often find themselves surviving, rather than truly succeeding or flourishing in health professions education. Here, we discuss the contrast between surviving and thriving and highlight the limitations of current support that often focuses on helping disabled students avoid crisis, rather than supporting flourishing. The idea of “surviving” speaks to a baseline existence where students are coping with the demands of a harmful system but are not provided with the means or opportunities to excel or fully participate in their university experience.

In her transcript, Samantha spoke at length of the difficulties she faced as a disabled student across university environments – formal, and informal; classroom, and social etc. She had accessed support at university and found this to be beneficial, but still, in the poem we have constructed from Samantha’s transcript, we see the purpose of support as perceived by Samantha as helping students to survive, rather than truly succeed at university.

My thing

Ya know
that’s my thing;
Struggling.

I’m trying
to make me
better – social,
but what if I can’t?

What if no matter
how much help I get
There is always
Struggle
Holding me back?

The repetition of “struggling” as “my thing” highlights how Samantha has internalised this experience as an inherent part of her identity within a university setting. We saw similar language in other participants’ transcripts – Husam, a multiply disabled learner, used variations of “push” and “pushing” to describe his actions and advocacy throughout his transcript, suggesting a constant effort – or struggle – to move forward that is part of his daily existence.

Further, the above poem questions the effectiveness of the support Samantha receives. She raised existential questions regarding whether struggle and difficulty will always be a part of her experience, regardless of the support on offer. Both Samantha and Husam shared with us a reality where the available support is only helpful in terms of maintaining a baseline of functionality, rather than fostering an environment where disabled students can excel. In order to excel, Samantha and Husam have to “push”, or “struggle” to navigate a system that is not designed to meet their needs.

## Discussion

Through creating poems from disabled students’ interviews on the topic of differential attainment, we have characterised their experiences in a way that not only describes barriers and successes, but embodies the emotional, human elements of their experience. Our poems speak to four themes: 1. The perseverance stereotype; 2. Managing the hidden curriculum; 3. Privilege and access; and 4. Surviving, not thriving. Such an embodied exploration, guided by our critical constructionist approach to poetic inquiry, has empowered us to explore: the impact of social structures, such as the support available for disabled students within higher education; culture, such as the hidden curriculum; and power, which exerts unequal effects on multiply minoritised students. Our poetic approach also makes explicit the emotions that accompany disabled students’ perceptions and experiences of DA. By integrating poems into our analysis, we hope this contribution will act to reconnect learners’ lived experiences with statistics reporting rates of DA, and descriptions of the nature of structural barriers learners face. This is important, because existing work is yet to capture the complexity of DA, and its significant impact on disabled students’ lives. By centering disabled learners’ experiences in our poems, we hope to advocate for more comprehensive and empathetic educational forms that create an environment where all students are provided with opportunities to flourish.

In exploring these experiences, one of the key themes we constructed concerned the negative power of stereotyping. Stereotypes are one way in which culture and power are communicated, and reproduced [26], and ableist stereotypes, which perpetuate narrow and often inaccurate perceptions of disability, are particularly harmful for disabled people [27]. This research highlights harmful stereotypes associated with the concept of “*perseverance*”, specifically the idea that life is a meritocracy, where sustained effort is all that is required to overcome obstacles. This fails to recognise the existence of well-documented systemic barriers, particularly for disabled people [28,29]. It implicitly suggests that failure to ‘overcome’ disability is a result of personal inadequacy rather than a consequence of broader social, institutional, and environmental factors. To our best knowledge, these stereotypes have not been discussed in relation to healthcare learners and this represents a novel contribution of our study, though there is literature recognising meritocracy as a myth [30], acknowledging ableist conceptions of “capability” within medical education [31], and troubling the focus on “individual” rather than “organisational” resilience [32]. Further research is required to fully characterise learners’ experience of this stereotype, its long-term impact, and connections to related concepts such as resilience.

Considering the stereotypes we communicate relating to “perseverance” in health professions education has important implications for assessment, given the association of this stereotype with differential attainment in this study. Addressing these stereotypes involves challenging dominant ideas regarding what constitutes competence and success for learners [31] and creating educational environments that are inclusive by design [33]. Designing, and redesigning assessments around the concept of “universal design” for learning can provide a practical way to create inclusive educational environments that accommodate the diverse needs of all learners. Universal design emphasises multiple modes and means of engagement, representation, action, and expression [34]. Instead of assisting individuals to address or circumvent barriers, energy is directed at designing or redesigning environments completely free of barriers for all learners [35]. Barriers will vary depending on educational context, but in this study, we heard stories of barriers to assessment including limited flexibility in assessment formats, rigid deadlines, and narrow definitions of success regarding what is assessed within a programme of study. Adopting universal design as a framework to address these issues may include creating multiple ways for students to engage with assessments (e.g., oral presentations, written assessments, digital portfolios), allowing students to choose the format best aligned with their needs [34].

The exploration of stereotypes in this study lays groundwork for understanding the challenges faced by disabled learners in accessing support and navigating HPE amidst systemic barriers. Our second theme, privilege and access, highlights disparities in how these systemic barriers are experienced and overcome and draws attention to the power of privilege in students’ ability to access necessary support and resources. As echoed in wider health professions education literature, our findings showcase intersectional complexities, in which student backgrounds and identities such as ethnicity, gender, immigration status, and socioeconomic status interact with disability [36,37] and significantly influence students’ experiences and access to support [38].

Interestingly, in addition to highlighting a lack of access to support services, and intersectional factors governing access, our findings go further to consider the lack of support for disabled students in navigating hidden curricula and culture. The poem constructed from Samantha’s interview, “My Thing”, communicates how she perceives support services’ purpose as supporting survival, rather than flourishing, whilst the poem constructed from Blake’s interview “Not this, but that”, communicates tensions in navigating disparities between what is said, and what is done by an institution. Literature in health professions education increasingly discusses the importance of “flourishing”, or “well-being characterised by positive emotion, engagement, strong relationships, meaning, and achievement” [39], which we see acknowledged by Samantha as critical, and lacking within disability support services. Blake’s poem relates to a lack of support for making sense of the “hidden curriculum”, well-known in medical education as the values, norms, beliefs, and practices not formally taught, but learned through immersion in an environment [40,41]. These tensions reveal critical gaps in support for disabled learners that need to be addressed. They highlight the necessity for institutions to move beyond basic compliance with accessibility standards and towards deeper, more meaningful engagement with the needs and aspirations of disabled students, ensuring that support structures are not only available but also effectively tailored to foster not just survival, but thriving in higher education.

Although offering specific interventions for addressing DA in disabled learners falls outside the scope of this article, we believe it is important to propose future directions for research and innovation. This is in line with our aim of building a comprehensive understanding of disabled learners’ experiences of DA experiences to guide evidence-based solutions. We recommend future research focus on applying the principles of universal design, as we have begun to discuss in this article [33], to assessment within health professions education to tackle DA. There is also a need for in-depth research regarding the effectiveness of existing support for disabled learners, including how to: improve availability; widen access; and ensure comprehensive approaches that promote learner wellbeing by supporting navigation of the hidden curriculum.

## Limitations

There are several limitations to this research. As a secondary data analysis, we did not control the questions asked within the original interview schedule, and so there are likely missed opportunities to deepen enquiry relating to disabled learners’ experiences of DA. We adapted Glesne’s [22] approach to poetic inquiry for secondary analysis, it would be beneficial for future research to utilise poetry within primary data collection settings, as fully aligned with Glesne’s method. With only a certain amount of space within this article, we can only present a subset of the poems we created. Though we selected those we felt representative of the themes we had constructed, and ensured spread of voice across participants, there is a loss of some participants’ voices in this process. Our sample offers broad representation of health professions, but there is likely need for future work to focus on specific contextual issues within-profession, also.

## Conclusion

Our poetic inquiry into the experiences of disabled students in relation to Differential Attainment in health professions education offers unique insight into the emotional, cultural, and contextual aspects of DA. In this way, poetry has helped us “set free” experiences of DA for disabled learners. Our study draws attention to the intersection of stereotypes, privilege, and systemic barriers, revealing a culture within health professions education that fails to acknowledge and address systemic barriers for disabled learners. The poems we have presented, within which we have tried hard to communicate each individuals’ embodied experiences of DA, emphasise the urgent need for more inclusive and responsive educational practices that go beyond basic compliance, aiming, instead, for a culture of true inclusivity and support. While this study does not propose specific interventions, it lays a critical foundation for future research, which should focus on characterising and improving support systems for disabled learners with the experience of these learners in mind.

## Additional File

The additional file for this article can be found as follows:

10.5334/pme.1392.s1 Supplementary Material. Semi-structured interview guide for Exploring Differential Attainment Through Realist Evaluation.

## Footnotes

The University of Manchester distinguish between home and international fee paying students. The rules about who pays ‘home’ fees are set by the Government’s Department for Education, and are described by the UK Council for International Student Affairs, UKCISA. They are detailed criteria, beyond scope for description here, but can be accessed at https://www.ukcisa.org.uk/Information—Advice/Fees-and-Money/England-HE-fee-status#layer-6082.

Ideally participants would have selected their own pseudonyms, but as this is a secondary analysis, we were limited in our approach.
