# Supplementary material for: Shut up, or Set Free: Poetic Inquiry into Disabled Students’ Experiences of Differential Attainment
Source: Perspect Med Educ. 2024 Nov 21;13(1):561–71. doi: 10.5334/pme.1392 (PMC11590743; doi:10.5334/pme.1392)
Supplement: Supplementary Material. — Semi-structured interview guide for Exploring Differential Attainment Through Realist Evaluation. [file pme-13-1-1392-s1.pdf]

Semi-structured interview guide for Exploring Differential Attainment Through Realist Evaluation

Students-generic questions

Introduction:

1. Introduction (offers your appreciation to the respondents for agreeing to participate in this research, re-introduce yourself and summarize the research and objectives).
2. The interviewee will have a summary of the research objectives and full contact address of the principal researcher. This includes a UoM email address.
3. Indicate how long the interview will take (60 to 90 minutes)
4. Provide a statement of confidentiality (this is an academic exercise; you will not be identified in the reports produced from this interview).
5. Confirmation of informed consent (I would like to confirm before we start that you are happy to proceed with the interview. During the interview if there is a question you are not comfortable to respond, you can skip it or indicate if you want to discuss it off record. Also, you can withdraw from the interview at any time you want to).
6. Check if the interviewee is happy to be recorded. If not, notes will be taken during the interview, and this will be noted on the consent form. I will remind those participants who chose to attend interviews via Zoom/Teams that they can turn off their camera if they wish so.
7. Make sure that the participants are comfortable during and immediately after the interviews. They should be advised to avoid issues that will disrupt their psychological wellbeing. Just in case if they become too emotional or tense, we will cut the interview short. If they will be seriously affected emotionally during the discussion, I will suggest that they can consider seeking support from the counselling services proposed on PIS and Debrief Sheet.

Interview Questions:

1. About yourself as student at UoM

a. Can you tell me about yourself, your subject, and the year of your study?

2. Attainment (a thing achieved, especially a skill or educational achievement)

a. Would you consider any aspect of your life that you just described to me an attainment? May you explain, please?

b. How do you evaluate your attainment at UoM? (By that I mean, how do you know how you are achieving and performing?)

c. How would you evaluate your attainment compared with that of your peers with different protected characteristics (e.g., WP, disability, ethnicity, religion, domicile/international, in care, ability etc.)?

d. (If *gap* raised in response to the question above) why do you think there is a gap in the attainments? Can you discuss contributing factors to this gap?

e. What are the facilitators and obstacles to your attainment? How can UoM counter the obstacles/ build awareness around them?

f. What is your ultimate goal studying at UoM? How do you try to achieve it?

g. How can UoM help you improve your attainment?

3. Experience at UoM

a) Why did you choose to study in UoM?

b) How do you describe your experience so far? How do you view your experience compared with that of your peers with different protected characteristics?

- about teaching, learning, and assessment
- about your relationship with teaching and Professional Support staff
- about your relationship with your peers
- about the HE culture (+in the UK for international students)
- about your engagement in social and community activities
- about your wellbeing
- about your future/career prospect
- accommodation and commute

c) What can be done to enhance your experience at UoM?

Conclusion

I have been asking all the questions so far; at this point I want to find out if you have any questions for me.

Thanks again for your time. I hope I can come back to you if I need more clarification on any issue.
